# Developing cancer warning statements for alcoholic beverages

**DOI:** 10.1186/1471-2458-14-786

**Published:** 2014-08-03

**Authors:** Simone Pettigrew, Michelle Jongenelis, Tanya Chikritzhs, Terry Slevin, Iain S Pratt, David Glance, Wenbin Liang

**Affiliations:** School of Psychology and Speech Pathology, Curtin University, GPO Box U1987, Perth, 6845 Australia; National Drug Research Institute, Curtin University, Perth, Australia; Cancer Council Western Australia, Perth, Australia; UWA Centre for Software Practice, University of Western Australia, Perth, Australia

**Keywords:** Alcohol, Warning labels, Prevention, Attitudes

## Abstract

**Background:**

There is growing evidence of the increased cancer risk associated with alcohol consumption, but this is not well understood by the general public. This study investigated the acceptability among drinkers of cancer warning statements for alcoholic beverages.

**Methods:**

Six focus groups were conducted with Australian drinkers to develop a series of cancer-related warning statements for alcohol products. Eleven cancer warning statements and one general health warning statement were subsequently tested on 2,168 drinkers via an online survey. The statements varied by message frame (positive vs negative), cancer reference (general vs specific), and the way causality was communicated (‘increases risk of cancer’ vs ‘can cause cancer’).

**Results:**

Overall, responses to the cancer statements were neutral to favorable, indicating that they are unlikely to encounter high levels of negative reaction from the community if introduced on alcoholic beverages. Females, younger respondents, and those with higher levels of education generally found the statements to be more believable, convincing, and personally relevant. Positively framed messages, those referring to specific forms of cancer, and those using ‘increases risk of cancer’ performed better than negatively framed messages, those referring to cancer in general, and those using the term ‘can cause cancer’.

**Conclusion:**

Cancer warning statements on alcoholic beverages constitute a potential means of increasing awareness about the relationship between alcohol consumption and cancer risk.

## Background

Following the successful introduction of warning labels on tobacco products, there have been increasing calls for similar warnings to be placed on alcoholic beverages [1–5]. This situation reflects the high costs associated with alcohol-related harms, which in Australia, are estimated to be almost $AU30 billion annually [6]. The calls for warning labels also reflect a growing evidence base relating to the relationship between alcohol consumption and a range of health problems including cancer, diabetes, cardiovascular disease, overweight and obesity, liver disease, fetal abnormalities, cognitive impairment, mental health problems, and accidental injury [7, 8]. Despite the demonstrated links between alcohol consumption and ill health, alcohol continues to be advertised heavily [9] and “as though it was not a toxic substance” [1]. Warning statements have been proposed as an important form of information provision to counteract the extensive promotion undertaken by the alcohol industry [10].

In a free-market economy it is assumed that consumers have access to the information they require to make informed choices [11]. This means that drinkers should have ready access to information relating to the possible negative consequences of their alcohol consumption [12, 13]. However, while there is growing awareness in the community of the dangers of heavy episodic drinking (i.e., ‘binge’ drinking) and drinking while pregnant, there is little understanding of the risks associated with alcohol consumption in general. In Australia, for example, around 90% of men and 81% of women believe they can drink alcohol every day without adverse health effects [14].

A particular knowledge deficit that has been identified relates to drinkers’ lack of awareness of the alcohol-cancer link [15, 16]. By comparison, they appear to have a better understanding of the relationship between alcohol consumption and other conditions such as liver cirrhosis, brain damage, mental illness, and heart disease [15]. As such, there is a need for effective product labelling as part of a comprehensive educational program to increase the likelihood that drinkers are aware of the increased risk of cancer associated with alcohol consumption. Even low levels of alcohol intake are implicated as a risk factor for a range of cancers [17, 18]. According to the International Agency for Research on Cancer [17], “Tumour types caused by drinking alcoholic beverages include cancers of the oral cavity, pharynx, larynx, oesophagus, liver, colorectum, and female breast.” It is estimated that 337 400 deaths per year worldwide are the result of alcohol-attributable cancers [17]. As the evidence base relating to the increased cancer risk posed by alcohol grows, there are increasing calls for cancer warning statements to be included on alcoholic beverage labels to increase public awareness of the alcohol-cancer link [16, 19]. Such warnings have the potential to be effective given high levels of fear of cancer in the general community [16, 20].

### Background

Warning labels on alcoholic beverages are now mandatory in several countries, including Brazil, Colombia, Costa Rica, France, Guatemala, Mexico, Russia, South Africa, Taiwan, Thailand, and the US [4]. The implemented labels have tended to focus on general health status (rather than specific health outcomes or diseases), drink driving, and the implications of drinking during pregnancy [12]. For example, in accordance with the Alcohol Beverage Labeling Act of 1988, the warning that has been in place on alcoholic beverages in the US since October 1989 states: “GOVERNMENT WARNING: (1) According to the Surgeon General, women should not drink alcohol beverages during pregnancy because of the risk of birth defects. (2) Consumption of alcoholic beverages impairs your ability to drive a car or operate machinery, and may cause health problems”.

Studies evaluating the effectiveness of the US warning label have concluded that while there have been some awareness, attitudinal, and recall effects, significant behavioral change has not eventuated [21–25]. This is in contrast to the experience with tobacco, where warning labels, as part of comprehensive programs involving a range of tobacco control strategies, have been successful in substantially lowering smoking rates [26]. One of the likely reasons for the lack of behavioral effects of alcohol warning labels in the US is their lack of prominence [25, 27]. Despite the legal requirement in the Act for warnings to be ‘located in a conspicuous and prominent place’, they have been found to have a low level of noticeability [28, 29]. This has been attributed to their location on the label, their orientation (they are often placed vertically), and the degree of clutter surrounding the message [30].

Another factor contributing to the reported lack of behavioral change may be the content of the messages that have been used to date and inevitable message wear-out. When first introduced, the US message had a stronger effect on awareness of the risks of drinking while pregnant relative to awareness of the dangers of drink driving, which was attributed to the former being relatively new information at the time [31]. After many years of exposure, the continuing emphasis on pregnancy and drink driving in the US warning may be consolidating existing knowledge, thereby producing very limited effects on awareness [32]. In addition, the information in the warning is general in nature and does not state specific health risks [13, 27].

Much of the limited previous work that has tested potential warning statements has focused on young people [3, 30, 33]. Further, very few studies have investigated the potential for cancer warnings on alcohol products to improve drinkers’ understanding of the relationship between alcohol consumption and cancer risk. In the small number of studies that have included cancer, the warning statements typically also referred to other diseases, preventing analysis of the effects of just the cancer message (e.g., GOVERNMENT WARNING: The consumption of this product, which contains alcohol, can increase the risk of developing hypertension, liver disease, and cancer [34]), or they reported only limited separate analyses of responses to the different types of messages included in the study [15].

Given the lack of research evidence relating to cancer warning messages, it is not known whether a more generalized cancer message (e.g., ‘Alcohol consumption increases the risk of cancer’) would be more or less convincing and motivating than a more specific cancer message (e.g., ‘Alcohol can cause breast cancer’). It is also not known whether warnings should be positively or negatively framed. Of the few studies examining message framing in the context of alcohol consumption, the focus has been on young people and the results have indicated that positively framed messages (e.g., ‘Make sure you are okay to drive ’) are preferred to negatively framed messages (e.g., ‘Drunk driving kills’) [3, 33, 35]. Further work is needed to determine which approach is likely to be most effective in risk communication among general adult populations.

In Australia, there is strong community support for the introduction of mandatory warning labels on alcoholic beverages [15, 30]. National reviews have recommended the introduction of warning labels on alcoholic beverages as one element in comprehensive approaches to reducing alcohol-related harm [36, 37]. However, it is acknowledged that there is little information available to guide the development of effective warnings [1, 4, 15], and this is especially the case in relation to cancer warnings. The aim of the present study was to develop and test a range of cancer warning statements that Australian drinkers consider believable, convincing, and personally relevant. The results can inform future policy efforts designed to provide drinkers with messages that have the potential to favorably influence their drinking behaviors.

## Methods

A multi-method approach was used to develop and test warning statements designed to advise drinkers of the cancer risk associated with alcohol consumption. Ethics clearance for the study was obtained from the University of Western Australia Human Research Ethics Committee and the research reporting process adhered to the RATS guidelines for reporting qualitative studies. Initially, focus groups were conducted with current drinkers to (i) explore perceptions of the harm associated with various levels of alcohol intake, (ii) assess awareness of the link between cancer and consumption, and (iii) workshop message content. On the basis of the qualitative findings, 12 warning statements were developed and then tested in a subsequent quantitative phase to determine the extent to which they may be effective in warning drinkers of the cancer risks associated with alcohol consumption. These two data collection stages are described further below.

### Qualitative phase

A social research agency used their respondent databases and random digit dialing to recruit drinkers to participate in the qualitative phase of the study. Forty-eight individuals who consumed at least 2–3 standard drinks of alcohol per month participated in six focus groups that were stratified by gender and age (18–30, 31–45, 46–64 years). The first author moderated all the groups using an exploratory and emergent approach. The groups commenced with a general discussion of the role of alcohol in Australian culture and in the participants’ lives. Motivations for and consequences of drinking were discussed, including any harms/diseases that participants considered to be associated with alcohol consumption. Perceptions of the potential for warning statements to be printed on alcoholic beverage containers were canvassed, and participants were invited to suggest wording for warning statements that could be effective in encouraging drinkers to reduce their intake. During this process, mock statements were constructed in line with the participants’ comments and used to stimulate further discussion. The statements generated during the focus groups included examples of wording that varied according to message framing (negative vs positive frame), strength of suggested causality (‘alcohol causes/can cause cancer’ vs ‘alcohol increases the risk of cancer’), types of cancer (general reference to cancer vs mentions of specific forms of cancer), and the use of the term ‘Warning’ (or not).

The groups ran from 60 to 92 minutes and were digitally audio-recorded. The recordings were transcribed and imported into NVivo9 software (QSR International, Pty Ltd) for coding and analysis. The coding schema included demographic variables and content nodes relating to the range of topics discussed in the groups. Examples of the latter included health-related beliefs about alcohol, types of drinking behaviors enacted, attitudes to cancer and its causes, types of message attributes mentioned, and perceptions of appropriate organizations/institutions to deliver cancer-related messages.

Throughout the focus group discussions, participants were primarily focused on the short-term effects of over-consumption and they frequently referred to their increased likelihood of engaging in other risky behaviors such as smoking and drink driving while intoxicated. There was also a clear understanding of the dangers associated with drinking while pregnant. The younger participants were additionally concerned about becoming over-emotional and being involved in accidents, acts of violence, and unprotected sex. Longer-term outcomes were less salient, but diseases such as liver problems, heart disease, cancer, and obesity were raised in most groups as potential outcomes from excessive alcohol consumption.

When the focus group discussions were directed to focus specifically on cancer risk, many participants appeared to believe that “everything gives you cancer”, and that alcohol consumed in moderation does not constitute a level of cancer risk that is worthy of concern. They therefore felt that it would be difficult to convince people that they needed to change their drinking habits on the basis of cancer risk. There was considerable discussion around the ideal wording for cancer warning statements that could be included on alcoholic beverage containers. Many felt that it would be more appropriate to refer to an increased risk of cancer (e.g., alcohol increases your risk of cancer), while others preferred a stronger causation message (e.g., alcohol causes cancer). Some participants were in favor of using high levels of fear to motivate behavioral change, and some suggested the provision of facts about the alcohol-related cancer to make the risk more tangible. Most felt that it would be more effective to nominate specific forms of cancer, rather than mentioning cancer in general, and to have multiple messages that rotate to ensure exposure to a relevant form of cancer (e.g., prostate cancer for men and breast cancer for women). There were some, however, who were in favor of generic cancer messages on the basis that they would apply to all drinkers. There were also mixed views on including the word ‘Warning’ at the front of the message. Some believed this would assist in attracting attention, while others argued that brief messages would work better and hence all extraneous words should be omitted.

On the basis of the qualitative findings and the existing evidence relating to the relationship between alcohol and cancer [17, 18], a series of 12 statements was developed that included examples of the attributes considered most relevant by the focus group participants. Table 1 lists the statements and their primary attributes. As the statements emerged from the qualitative data, there was coverage of each the primary attributes, but no attempt was made to equalize the number of messages exhibiting each attribute. Instead, the statements considered most motivating and that were best supported by the existing literature were carried through to the next stage of data collection.

**Table 1:** **Warning statements by primary characteristics**

Statement	Message frame	Numerical evidence	Fear appeal*	Specificity of cancer
Warning: alcohol harms your health	Negative			
Warning: alcohol increases your risk of cancer	Negative		Yes	General
Alcohol causes cancer: reduce your intake to reduce your risk	Positive			General
Reduce your drinking to reduce your risk of cancer	Positive			General
Alcohol increases your risk of bowel cancer	Negative		Yes	Specific
Alcohol increases your risk of breast cancer	Negative		Yes	Specific
Alcohol increases your risk of breast, bowel, throat, and mouth cancer	Negative		Yes	Specific
Alcohol increases your risk of cancer	Negative		Yes	General
Alcohol can cause breast cancer	Negative		Yes	Specific
Alcohol can cause bowel cancer	Negative		Yes	Specific
Alcohol causes around 5000 new cases of cancer each year	Negative	Yes		General
Alcohol causes 1 in 20 cancer deaths	Negative	Yes		General

*Cancer warning statements that did not feature a positive frame or numerical evidence were classified as fear appeal messages.

### Quantitative phase

An online survey of Australian drinkers (*n* = 2,168) was used to measure the believability, convincingness, and perceived personal relevance of the 12 warning statements. A large web panel provider was used to access the sample and disseminate the online questionnaire. To be eligible to participate in the study, respondents had to be at least 18 years of age (the legal age to purchase alcohol in Australia) and consume alcohol at least two to three times per month. Quotas were used to generate a sample that was roughly equivalent to the Australian adult population in terms of key demographic attributes. Table 2 provides the sample profile.

**Table 2:** **Sample profile (n = 2,168)**

Sample characteristics		%
Gender	Male	50.5
	Female	49.5
Age	< 31 years	42.7
	31 – 45 years	26.4
	46 – 65 years	30.9
Harm profile*	Low ST/low LT risk	19.7
	High ST/low LT risk	49.2
	Low ST/high LT risk	1.0
	High ST/high LT risk	30.2
Education	Tertiary qualification	42.9
	Non-tertiary	57.1

*As per NHMRC alcohol consumption guidelines [8].

Of the 12 statements included in this phase of the research, one was a general health message (‘Warning: Alcohol harms your health’) that was used as a comparison for the 11 cancer warning statements that were the primary focus of the study. Along with a series of demographic (e.g., age, gender, education), behavioral (e.g., amount and frequency of alcohol consumption), and attitudinal questions (e.g., quantity and frequency of alcohol consumption, perceptions of the healthiness of alcohol), respondents were randomly exposed to three of the 12 statements. The statements were presented as plain text, and were not incorporated into label designs to avoid contamination effects from other label elements. After exposure to each statement, respondents were asked to report the extent to which they found the message to be believable, convincing, and personally relevant [38, 39]. Respondents thus responded to each statement individually, and statement order was randomly assigned to minimize order effects.

#### Analysis

Descriptive analyses were initially conducted to calculate the believability, convincingness, and personal relevance scores for each of the 12 statements. Tests for normality (skewness and kurtosis) were also conducted prior to any inferential analysis. These tests revealed that the variables under investigation were all normally distributed (skewness < 2.00, kurtosis < 4.00).

The factors influencing believability, convincingness, and personal relevance scores across the 11 cancer statements were then examined using hierarchical multiple linear regression. Factors entered as independent variables were gender (male vs female), age, type of alcohol consumed most often (beer, wine, spirits), and education level (tertiary, non-tertiary). In addition, the Australian National Health and Medical Research Council (NHMRC) [8] alcohol guidelines were used to allocate respondents to one of four harm profiles. According to the guidelines, “no more than two standard drinks on any day reduces the lifetime risk of harm from alcohol-related disease or injury” and “no more than four standard drinks on a single occasion reduces the risk of alcohol-related injury arising from that occasion”. The four profiles were therefore constructed as follows: *low short-term/low long-term risk* – no more than four drinks in a single sitting over the previous 12 months and an average consumption level of two or fewer standard drinks per day; *high short-term/low long-term risk* – more than four drinks in a single sitting and an average daily intake of two drinks or fewer; *low short-term/high long-term risk* – no more than four drinks in a single sitting and an average daily intake of more than two drinks; and *high short-term/high long-term risk* – more than four drinks in a single sitting and an average daily intake of more than two drinks.

Regression analyses were accomplished in two steps. First, separate univariate regression analyses were conducted for each possible predictor to avoid any complications due to multicollinearity [40]. In the second step, significant univariate predictors were included in a simultaneous multivariate regression model to determine the unique contribution of each significant predictor. The assumption of no multicollinearity was satisfied in all multivariate regressions conducted, with all independent variables associated with a tolerance level above the minimum criterion of 0.20 and a variance inflation factor below the maximum criterion of 10. A *p* value of < .05 was used as the significance cut-off.

To assess whether specific aspects of the statements under investigation were related to outcomes, the 11 cancer statements were clustered according to key message attributes. Paired-samples *t*-tests were then used to compare believability, convincingness, and personal relevance across these various attributes.

## Results

As each respondent was exposed to three of the 12 statements, the sample size for each statement was approximately one-quarter of the total sample (M = 540, range 534–548). Table 3 shows the list of statements that were analyzed individually and ranked by their believability, convincingness, and personal relevance scores. The highest scoring statement across all three outcomes was the general health warning message.

**Table 3:** Means and standard deviations for believability, convincingness, and personal relevance by individual statement*

Statement	Believability	Convincingness	Personal relevance
Warning: alcohol harms your health (n = 547)	3.68 (1.11)	3.42 (1.17)	2.91 (1.23)
Alcohol increases your risk of bowel cancer (n = 541)	3.34 (1.13)	3.20 (1.13)	2.86 (1.17)
Alcohol causes cancer: reduce your intake to reduce your risk (n = 541)	3.26 (1.20)	3.14 (1.22)	2.76 (1.19)
Alcohol increases your risk of breast, bowel, throat, and mouth cancer (n = 544)	3.26 (1.20)	3.09 (1.22)	2.82 (1.20)
Reduce your drinking to reduce your risk of cancer (n = 545)	3.24 (1.18)	3.10 (1.18)	2.77 (1.22)
Alcohol increases your risk of cancer (n = 535)	3.24 (1.23)	3.09 (1.25)	2.83 (1.27)
Warning: alcohol increases your risk of cancer (n = 548)	3.24 (1.22)	3.07 (1.21)	2.86 (1.24)
Alcohol can cause bowel cancer (n = 543)	3.23 (1.14)	3.05 (1.14)	2.79 (1.18)
Alcohol causes around 5000 new cases of cancer each year (n = 541)	3.09 (1.18)	2.97 (1.19)	2.64 (1.23)
Alcohol causes 1 in 20 cancer deaths (n = 540)	3.00 (1.22)	2.95 (1.23)	2.64 (1.23)
Alcohol increases your risk of breast cancer (n = 545)	2.98 (1.25)	2.86 (1.23)	2.48 (1.31)
Alcohol can cause breast cancer (n = 534)	2.89 (1.25)	2.78 (1.23)	2.37 (1.25)

*Five-point scale: 1 (not at all believable/convincing/relevant) to 5 (very believable/convincing/relevant).

A check of order allocation effects revealed that of the 36 scores across the 12 messages and the three outcome variables of believability, convincingness, and personal relevance, there were no significant order effects for 34 of the scores. Of the two scores that were affected, the order effects were in opposite directions, with one showing a higher score when the message was shown first and one showing a lower score. It thus appears that prior exposure to statements did not prime participants and influence responses to later-presented messages.

Table 4 provides the significant results from the multivariate hierarchical regression analyses that were conducted after initial univariate analyses identified respondent attributes that were significantly associated with the outcome variables without the influence of multicollinearity. Health beliefs relating to alcohol were controlled for in Block 1 of the regression analysis. All other respondent attributes were included in Block 2. The 11 cancer statements were first analyzed in aggregate, followed by clustering to permit comparisons according to message characteristics (as shown in Table 1).

**Table 4:** **Hierarchical multiple regression results (Block 2)**

	Believability (Δ***R*** ^2^ = .01; ***R*** ^2^_adjusted_ = .064)						Convincingness (Δ***R*** ^2^ = .02; ***R*** ^2^_adjusted_ = .064)						Personal relevance (Δ***R*** ^2^ = .06; ***R*** ^2^_adjusted_ = .147)					
Predictor	***b***	***SE***	95% CI for ***b***	β	***p***	Part ***r*** ^2^	***b***	***SE***	95% CI for ***b***	β	***p***	Part ***r*** ^2^	***b***	***SE***	95% CI for ***b***	β	***p***	Part ***r*** ^2^
Overall, what is your attitude or opinion about alcohol consumption	-.10	.03	-.16, -.05	-.08	< .001	-.07	-.09	.03	-.15, -.03	-.07	.002	-.07	-.08	.03	-.14, -.03	-.06	.004	-.06
How harmful or beneficial do you think your current alcohol consumption is to your health	-.17	.03	-.22, -.12	-.15	< .001	-.14	-.17	.03	-.22, -.12	-.14	< .001	-.14	-.28	.03	-.33, -.23	-.23	< .001	-.21
Do you consider alcohol to be part of a healthy diet	-.10	.02	-.14, -.05	-.10	< .001	-.09	-.10	.02	-.15, -.06	-.10	< .001	-.09	-.04	.02	-.08, .01	-.04	.107	-.03
Gender	.09	.05	.00, .18	.04	.058	.04	.19	.05	.09, .29	.09	< .001	.08	.32	.05	.23, .42	.14	< .001	.14
Age	.00	.00	-.01, .00	-.05	.014	-.05	-.01	.00	-.01, .00	-.06	.003	-.06	-.01	.00	-.01, .00	-.08	< .001	-.08
Tertiary education	-.20	.05	-.29, -.11	-.09	< .001	-.09	-.12	.05	-.22, -.03	-.06	.011	-.06	-.14	.05	-.23, -.04	-.06	.004	-.06
Alcoholic beverage							-.12	.03	-.18, -.06	-.09	< .001	-.08	-.20	.03	-.26, -.14	-.14	< .001	-.13
Harm profile													.18	.02	.13, .22	.18	< .001	.16

Across the 11 cancer statements, age and tertiary education were related to believability, with younger respondents and those with a tertiary qualification finding the statements more believable than older and less educated respondents. The results were similar for convincingness, with age and tertiary qualifications found to be significant predictors. In addition, females and beer and wine drinkers were likely to find the messages more convincing than were males and those who most frequently consumed spirits. Finally, females, younger respondents, those in the high short-term/high long-term harm profile category, beer and wine drinkers, and those with a tertiary education were more likely than other respondents to find the messages personally relevant.

### Outcomes by drinking status

Of interest were the outcomes for those drinkers who are most at risk of alcohol-related harm, specifically those in the high short-term/high long-term risk category. Scores obtained for believability, convincingness, and personal relevance by those in this category were therefore compared to the rest of the sample. An independent samples *t*-test revealed no significant difference in believability or convincingness, but the high-risk drinkers were more likely than other respondents to consider the statements personally relevant (M = 2.58 vs 2.99: *t*(2166) = -7.90, *p* < .001, *d* = -0.36).

When hierarchical regression analyses were repeated for those respondents in the highest risk category, the tertiary education, gender, and beverage type differences for statement believability and convincingness evident in the total sample no longer applied. Age remained a significant predictor, with younger high-risk drinkers finding the messages more believable and convincing than older high-risk drinkers. Consistent with the analyses conducted on the entire sample, the personal relevance of the statements among high-risk drinkers was significantly higher for females, younger respondents, those with tertiary qualifications, and those preferring beer and wine (results not shown).

### Outcomes by message attributes

To assess whether specific aspects of the statements were related to outcomes, the 11 cancer warning statements were clustered according to key message attributes and paired samples *t*-tests were used to compare outcomes by attribute. These attributes included primary message characteristics, causation wording, and general versus specific references to cancer.

#### Primary message characteristics

Two statements were categorized as featuring a positive message frame (because they offered positive actions to be undertaken to reduce risk), two statements were categorized as featuring numerical evidence, and the remaining statements were categorized as featuring a fear appeal. While technically all the statements utilized fear appeals because of their function as warnings, those statements allocated to the fear appeal category were those that did not feature another dominant message characteristic and hence were labeled simply as fear appeals to facilitate differentiation.

The means presented in Table 5 show that statements characterized by a positive message frame were considered more believable than statements that primarily used fear appeals (*t*(875) = 4.29, *p* < .001, *d* = 0.10) and numerical evidence (*t*(365) = 2.99, *p* = .003, *d* = 0.12). The same trend in results was found for convincingness and personal relevance. However, when all outcome variables were examined further, the difference between positive message frame and numerical evidence was only evident among males (*t*(199) = 2.51, *p* = .013, *d* = 0.14). Additionally, the difference in personal relevance between positive message frame and fear appeal was significant in males only (*t*(468) = 7.75, *p* < .001, *d* = 0.29). Although a significant difference was found between fear appeal and numerical evidence in terms of personal relevance for both males and females, the direction of this effect differed between genders, with males finding statements featuring numerical evidence more personally relevant than those using a fear appeal (*t*(438) = 2.55, *p* = .011, *d* = 0.09) and females finding the opposite (*t*(441) = 4.02, *p* < .001, *d* = 0.11).

**Table 5:** **Believability, convincingness, and personal relevance scores by statement characteristics**

Statement characteristic	Believability mean (SD)	Convincingness mean (SD)	Personal relevance mean (SD)
*Message type*			
Positive frame (n = 978)	3.23 (1.18)^a^	3.10 (1.19)^a^	2.76 (1.20)^a^
Fear appeal (n = 2055)	3.15 (1.14)	3.01 (1.15)	2.68 (1.15)
Numerical evidence (n = 986)	3.05 (1.19)	2.96 (1.21)	2.63 (1.23)
*Message wording*			
‘Increases risk of cancer’ (n = 994)	3.15 (1.19)^b^	3.02 (1.18)^b^	2.66 (1.23)
‘Can cause cancer’ (n = 989)	3.06 (1.19)	2.92 (1.19)	2.58 (1.22)
*Cancer specificity*			
General (n = 1964)	3.18 (1.15)^c^	3.05 (1.16)^c^	2.76 (1.20)^c^
Specific (n = 1817)	3.12 (1.16)	2.99 (1.16)	2.64 (1.18)

^a^Mean for positive frame message type significantly different to mean for fear appeal and numerical evidence message types.

^b^Mean for ‘increases risk’ wording significantly different to mean for ‘can cause risk’ wording.

^c^Mean for general statements significantly different to mean for specific statements.

#### Causation wording

To assess whether the way in which causation was stated affected outcomes, comparisons were made between those statements that were identical in all other respects. The scores for ‘Alcohol increases your risk of bowel cancer’ and ‘Alcohol increases your risk of breast cancer’ were therefore compared with the scores for ‘Alcohol can cause bowel cancer’ and ‘Alcohol can cause breast cancer’ respectively. As shown in Table 5, the two statements featuring ‘increases risk’ wording were considered more believable than the equivalent ‘can cause’ statements (*t*(363) = 2.86, *p* = .004, *d* = 0.12), although when examined further this difference was only evident among females (*t*(183) = 2.21, *p* = .028, *d* = 0.13). The ‘increases risk’ wording was also more convincing across the sample than the ‘can cause’ wording (*t*(363) = 3.55, *p* < .001, *d* = 0.14), but no significant differences for personal relevance were found between wording types (*t*(363) = 0.65, *p* = .514, *d* = 0.03).

#### General versus specific references to cancer

Outcomes for statements referring to a specific type of cancer were compared to those statements referring to cancer in general (Table 5). Overall, the general cancer statements were found to be more believable (*t*(1612) = 3.75, *p* < .001, *d* = 0.07), convincing (*t*(1612) = 4.32, *p* < .001, *d* = 0.07), and personally relevant (*t*(1612) = 6.84, *p* < .001, *d* = 0.13) than the specific cancer statements.

## Discussion

The aim of this study was to develop a range of cancer warning statements for the purpose of informing alcohol labelling policies. During an initial qualitative phase, focus group participants expressed a preference for brief, concise statements, an outcome that is consistent with previous research highlighting the need for warning labels to simply and directly communicate the adverse consequences of contra-indicated behaviors [41]. On the basis of the focus group findings, a series of statements that varied according to message characteristics was developed for further testing. In the quantitative phase of the study, one general health warning statement and 11 cancer-related warning statements were tested with a large sample of Australian drinkers. The general health warning received the highest believability, convincingness, and personal relevance scores. This outcome is in line with greater pre-existing knowledge in the community relating to the general health risks associated with alcohol consumption relative to specific knowledge relating to cancer risk [15, 31].

Overall, responses to the cancer messages were neutral to favorable, indicating that they are unlikely to encounter high levels of negative reaction from the community if introduced on alcoholic beverages. This finding is consistent with reported high levels of support for mandatory warnings on alcohol products in Australia [15, 30]. There were some significant differences in the outcome variables by respondent and message characteristics. Females, younger respondents, and those with higher levels of education generally found the statements to be more believable, convincing, and personally relevant. Positively framed statements (i.e., those that focus on the gains to be obtained from engaging in the recommended behavior [42]), those referring to specific forms of cancer, and those using ‘increases risk of cancer’ performed better than negatively framed statements, those referring to cancer in general, and those using the term ‘can cause cancer’.

The results demonstrating preference for positively framed messages are consistent with those of previous warning message research that has focused on messages targeting younger drinkers [3, 33, 35, 43] and smokers [44, 45]. However, of note is that while on aggregate the positively framed messages received higher attitudinal scores, the individual statement ‘Alcohol increases your risk of bowel cancer’ (a negatively framed message) performed the best of all the cancer messages. The relationship between message type and attitudinal response may therefore be complex and involve numerous other factors. Previous research has suggested that such factors may include prior knowledge [46] and perceived self-efficacy [47]. Comparisons with previous research investigating the efficacy of messages using different cancer-related wording are not possible due to a lack of prior work in this area.

In practical terms, the differences according to message and respondent characteristics were minor and the proposed statements could be feasibly used in rotation, as has been implemented in the case of tobacco warning labels and recommended for alcohol warnings [4]. The lower scores attributed to the breast cancer statements are worthy of note. While the lower levels of personal relevance are understandable in the context of this form of cancer primarily afflicting women, the outcomes relating to believability and convincingness are likely to be at least partially the result of the well-publicized genetic causes of breast cancer [48]. A strong belief in genetic causes may prevent individuals from appreciating the role of alcohol in contributing to risk for this form of cancer. Rather than making messages relating to breast cancer less relevant, pre-existing beliefs may make them all the more important to promote understanding of how lifestyle decisions can reduce risk.

The results relating to heavy drinkers are especially encouraging. Those drinking an average of more than two standard alcoholic drinks per day and more than four drinks in a single sitting are at higher short- and long-term risk of alcohol-related harm [8]. This group found the messages to be no less believable or convincing than those consuming alcohol at lower risk levels, but they were more likely to consider the messages personally relevant. This may serve to alleviate concerns about potential psychological reactance outcomes among heavy drinkers that could result in even higher levels of consumption.

This study has several limitations. In the first instance, respondents were exposed to three statements in total, which may have influenced overall responses. The random allocation of three statements out of a possible 12 and the random order of exposure within the three statements will have minimized order effects for individual statements, but future studies may seek to limit exposure to one message per respondent to address this limitation. Second, the use of a web panel and non-representative sampling (e.g., non-drinkers and very light drinkers were not included) constitute a further limitation. However, the large sample size and coverage of major demographic groups assists in addressing this concern. Future research could consider different forms of recruitment to potentially achieve a more representative sample, although presentation of warning statements during study administration is likely to require either a print or online format to replicate the on-package location of such messages. Third, the use of plain text statements does not necessarily provide insight into the effects that may be observed once the warnings are incorporated into labels on beverage containers. Now that potential statements have been developed and tested, the next stage is to engage in further testing in the context of actual and/or mock product labels.

Finally, the present study was largely limited to analyses of drinkers’ perceptions of the believability, convincingness, and personal relevance of a range of cancer-related alcohol warning statements. Further work is needed to assess the ability of such messages to change behavioral intentions and stimulate behavioral change. However, it is unrealistic to expect warning labels to have substantial effects at the population level without integration with a range of other alcohol control measures. As in the case of tobacco, it is likely that the best outcomes will be achieved by implementing a complementary suite of strategies that in combination can modify individual behaviors and social norms [4, 49]. Further research is therefore needed to investigate the optimal combination of strategies to achieve this objective. In the interim, the warning statements developed and tested in the present study offer a starting point for policy makers seeking guidance on potentially effective warning statement attributes.

## Conclusion

A growing evidence base relating to the long-term harms associated with alcohol consumption is resulting in calls for more comprehensive approaches to harm minimization. In particular, increased evidence of the alcohol-cancer link and low levels of community awareness of this link highlight the need for greater public education on this issue. Cancer warning statements on alcoholic beverages constitute a potential means of increasing awareness about the relationship between alcohol consumption and cancer risk. The results of the present study indicate that such statements are likely to be considered believable, convincing, and personally relevant by the Australian drinking public.
